# Lack of Association between Pulse Steroid Therapy and Bone Mineral Density in Patients with Multiple Sclerosis

**DOI:** 10.1155/2016/5794910

**Published:** 2016-02-04

**Authors:** Serap Zengin Karahan, Cavit Boz, Sevgi Kilic, Nuray Can Usta, Mehmet Ozmenoglu, Vildan Altunayoglu Cakmak, Sibel Gazioglu

**Affiliations:** Karadeniz Technical University, 61080 Trabzon, Turkey

## Abstract

Multiple sclerosis (MS) has been associated with reduced bone mineral density (BMD). The purpose of this study was to determine the possible factors affecting BMD in patients with MS. We included consecutive 155 patients with MS and 90 age- and sex-matched control subjects. Patients with MS exhibited significantly lower* T*-scores and* Z*-scores in the femoral neck and trochanter compared to the controls. Ninety-four (61%) patients had reduced bone mass in either the lumbar spine or the femoral neck; of these, 64 (41.3%) had osteopenia and 30 (19.4%) had osteoporosis. The main factors affecting BMD were disability, duration of MS, and smoking. There was a negative relationship between femoral BMD and EDSS and disease duration. No association with lumbar BMD was determined. There were no correlations between BMD at any anatomic region and cumulative corticosteroid dose. BMD is significantly lower in patients with MS than in healthy controls. Reduced BMD in MS is mainly associated with disability and duration of the disease. Short courses of high dose steroid therapy did not result in an obvious negative impact on BMD in the lumbar spine and femoral neck in patients with MS.

## 1. Introduction

Multiple sclerosis (MS), a chronic progressive demyelinating disease of the central nervous system, is the most common cause of acquired disability in young adults [1]. Several previous studies, but not all, have shown reduced bone mineral density (BMD) in patients with MS, indicating that patients with MS have a higher risk for osteoporosis [2–10]. The exact mechanism and causes of the reduced BMD in MS patients are as yet unclear.

MS itself causes problems with vision, balance, coordination, and motor performance, all of which increase the risk of osteopenia, osteoporosis, and fractures. Genetic factors, corticosteroid use, the presence of various cytokines involved in the pathogenesis of MS, and calcium and vitamin D deficiency have also been implicated as plausible etiopathogenic mechanisms leading to secondary osteoporosis in patients with MS [4, 5, 10, 11].

Chronic use of glucocorticosteroids has been shown to increase bone resorption and to reduce bone formation and is a risk factor for osteoporosis [12, 13].

High-dose methylprednisolone is the gold standard in pharmacological management of MS relapses. The effect of high-dose methylprednisolone pulses on BMD in MS is still controversial, however. There are some concerns over possible harmful effects of steroid pulse on bone composition in patients with MS.

Understanding the role of pulse steroid in bone status will help with planning optimal relapse management in MS. The purpose of this study was to determine the possible factors affecting bone mineral density in patients with MS.

## 2. Subjects and Methods

One hundred fifty-five (51 males and 104 females) consecutive patients with MS diagnosed according to McDonald 2005 criteria [14] regularly followed up in our outpatient MS clinic and 90 age- and sex-matched control subjects were included in the study. The protocol was approved by the university local ethical committee, and all individuals gave informed consent. Subjects with a medical condition or medication use that might influence bone mineral metabolism such as menopause, hyperthyroidism, rheumatoid arthritis, hormone replacement therapy, or use of long-term oral contraceptive exceeding 1 year were excluded from the study. Subjects with body mass index higher than 35 kg/m^2^ were also excluded. None of the case or control individuals were receiving calcitonin, vitamin D, bisphosphonates, or selective estrogen receptor modulators. All patients were in remission and had not received pulse intravenous methylprednisolone for at least 1 month during the BMD measurements.

Neurological examination and Expanded Disability Status Scale (EDSS) calculation were performed by a neurostatus EDSS-certified neurologist. EDSS steps 0 to 4.5 refer to patients who are fully ambulatory, while EDSS steps 5.0 to 9.5 are defined by impairment in ambulation [15]. For this study we defined MS subgroups as a fully ambulatory group (EDSS < 5) and an impaired ambulation group (EDSS ≥ 5).

Treatment of MS relapse at our center consists of 3–7 days of 1000 mg intravenous methylprednisolone without oral steroid taper.

The control group consisted of randomly selected healthy individuals representing friends of patients in the neurology clinic or hospital staff who consented to participate for research purposes.

Dual energy X-ray absorptiometry (DEXA) was used to measure BMD values at the lumbar spine (L1–L4) and femoral neck and trochanter. The scans and data analysis were performed by the same technician.

BMD results were expressed as g/cm^2^, in addition to *T*-score and *Z*-score. *T*-score is used to make a diagnosis of normal bone density, osteoporosis, or osteopenia according to the classification system of World Health Organization (WHO) [16]. Normal BMD was defined as *T*-score of −1.0 or above, osteopenia was defined as *T*-score less than −1.0 and greater than −2.5, and osteoporosis was defined as *T*-score of −2.5 or less.

Biochemical parameters which may have an impact on BMD, serum 25-hydroxyvitamin D_3_, intact parathyroid hormone, calcium, phosphorus, and total alkaline phosphatase were measured.

### 2.1. Statistical Analyses

Continuous data were presented as means (± standard deviation) and categorical data were presented as numbers. Comparison of groups was performed using *χ* ^2^ test for dichotomous variables and independent-samples 2-tailed *t*-tests for continuous variables. Stepwise multiple regression analysis was performed to examine factors independently associated with *Z*-scores in the three regions. Gender, age, MS duration, BMI, EDSS, MS type, cumulative steroid administration, and smoking habits were included in the model and assumed to be independent variables. Relations between BMD and numerical data (age, disease duration, cumulative steroid dose, and vitamin D level) were evaluated using Pearson's correlation analysis. Spearman's rank correlation coefficients (*r*) were calculated to determine the relationship between *Z*-scores for BMD and EDSS. All tests were performed using SPSS (version 13, SPSS, Chicago, IL), and *p* < 0.05 was considered statistically significant.

## 3. Results

Descriptive characteristics of patients with MS and controls are shown in Table 1. No statistically significant differences were found in terms of age, sex ratio, smoking duration, or BMI between the MS patients and controls. The mean ages of male and female patient subgroups were also similar to those of the male and female controls.

Mean age of the entire MS patient group was 35.6 (18–55), and mean EDSS was 2.6 (0–7.5). One hundred twelve patients with MS were fully ambulatory (EDSS score < 5) and 43 had limited ambulation (EDSS ≥ 5).

Of these MS patients, 110 (71%) had relapsing-remitting multiple sclerosis, 34 (22%) had secondary progressive multiple sclerosis, and 11 (7%) had primary progressive multiple sclerosis. The majority of patients (135 of the 155) had received pulse methylprednisolone therapy prior to enrolment, with an average cumulative dose of 13.9 g.

One hundred five of the patients with MS used disease-modifying drugs (75 used interferon beta, 24 used glatiramer acetate, and 6 used natalizumab).

Table 2 shows BMD assessment at the three regions of interest in the patients and controls. Mean BMD measurements did not exhibit any significant difference at the lumbar site in the female and male patient groups compared to the controls. Male and female patients exhibited significantly lower *T*-score and *Z*-score compared to the controls in the femoral neck and trochanter. BMD score expressed as g/cm^2^ was significantly lower at the femoral neck and trochanter in female MS patients than in the female controls.

**Figure 1:**
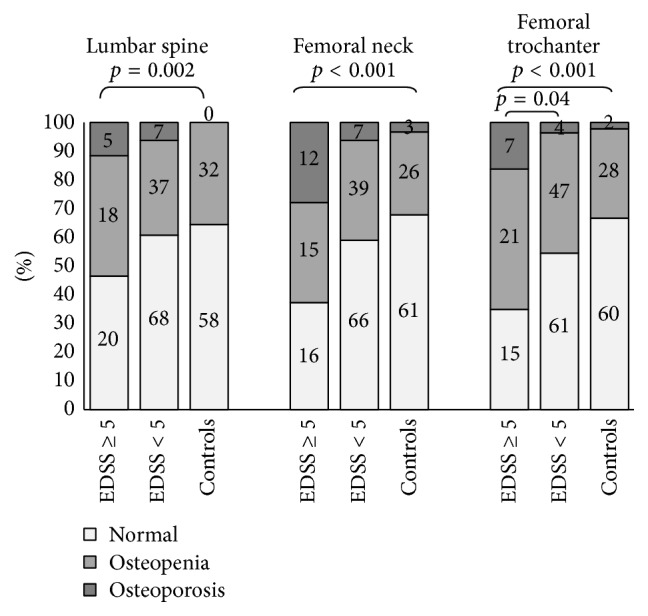
Count and proportions of patients and controls with WHO classified 12 osteoporosis (*T*-score ≤ −2.5), osteopenia (−2.5 < *T*-score ≤ −1.0), and normal (*T*-score > −1). *p* value was calculated using *χ* ^2^ test and refers to patients with osteopenia or osteoporosis in at least one site compared to controls. Count of the patients and controls in each group is shown on the bars.

The numbers and proportions of patients and controls with WHO classified osteoporosis and osteopenia at the three regions are shown in Figure 1. Ninety-four (60%) of the patients with MS had either osteoporosis or osteopenia in at least one measured site. In fully ambulatory patients (EDSS < 5), the proportions of osteopenia and osteoporosis did not differ significantly at the lumbar spine (*p* = 0.10) or femoral neck (*p* = 0.21), but the proportion of osteopenia was significantly higher at the femur trochanter (*p* = 0.04).

In limited ambulatory MS patients (EDSS ≥ 5), the proportions of osteopenia and osteoporosis were significantly higher at all three regions of interest. Only 13 of the 43 (30%) of the limited ambulation patients had normal BMD measurements at all regions.

Multiple linear regression analyses showed an independent negative association between *Z*-scores and smoking, EDSS at the femoral neck (*p* = 0.019 and 0.005, resp.), and trochanter (*p* = 0.049 and 0.03, resp.) and age at the femoral neck (*p* = 0.016). Disease duration was associated with reduced BMD at the lumbar site (*p* = 0.02). Cumulative steroid dose and relapse rate were not associated with BMD scores at any region (Table 3).

**Figure 2:**
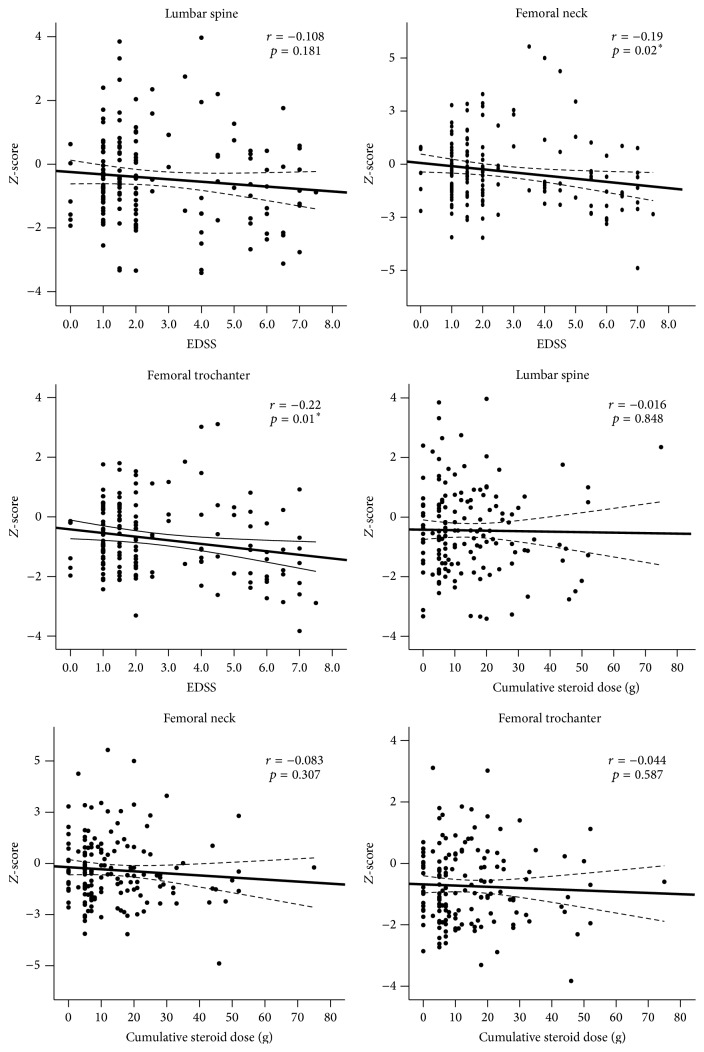
Associations between bone *Z*-scores with EDSS scores or cumulative steroid dose. Least-squares lines and 95% confidence interval are shown. *∗* indicates that correlation is significant at the 0.05 level (2-tailed).

Correlation analyses revealed a negative association of *Z*-scores with EDSS at the femoral neck (*r* = −0.19 and *p* = 0.02) and the trochanter (*r* = −0.22, *p* = 0.01), but there was no significant association at the lumbar region. There was no correlation between cumulative steroid dose and BMD values at any region (Figure 2). Distributions of *Z*-scores in relation to disability and cumulative steroid dose are shown in Figure 2.

Levels of vitamin D intact parathyroid hormone, calcium, phosphorus, and total alkaline phosphatase were not significantly correlated with BMD measurements.

MS duration was significantly negatively correlated with *Z*-scores at the femoral neck (*r* = −0.16 and *p* = 0.046) and femoral trochanter (*r* = −0.18 and *p* = 0.025). Length of smoking status was significantly correlated with *Z*-scores in the lumbar region (*r* = −0.402 and *p* = 0.008), femoral neck (*r* = −0.45 and *p* = 0.002), and femoral trochanter (*r* = −0.43 and *p* = 0.005).

## 4. Discussion

This study demonstrated that male and female patients with MS have significantly higher prevalence of osteoporosis and osteopenia at femoral regions, but not at the lumbar site, compared with healthy age-matched control subjects. The same results have been reported in previous studies [9, 10, 16–18] for the femoral site; however, other studies have also reported decreased BMD at the lumbar site [6, 10, 19].

Our major findings in this study are that reduced BMD in patients with MS is mainly associated with higher EDSS scores and that short courses of high-dose methylprednisolone therapy in MS relapses did not result in any obvious negative impact on BMD.

The deleterious effect of low-dose, long-term oral steroid treatment on bone homeostasis is well known [20]. Epidemiological studies have shown that fracture risk increases rapidly after commencement of oral steroid treatment and is related to the dose and duration of steroid exposure [21]. However, it has been reported that the fracture risk tends to return toward baseline levels after discontinuation of steroids [22].

In agreement with our study, previous studies have shown that intermittent intravenous high-dose methylprednisolone commonly used as a therapy for acute MS relapses does not affect BMD [9, 23–26], but others have reported a negative association between steroid therapy and bone status [18, 19, 27, 28]. Cosman et al. determined suppression of bone formation in patients with MS who received 1 g IV MP for 10 days followed by 28-day oral glucocorticoid [19]. Sioka et al. also failed to demonstrate a statistically significant correlation between BMD in all investigated sites and total steroid dose (only 20 g on average) and number of relapses. However, that study did demonstrate a significantly increased frequency of osteopenia or osteoporosis when WHO diagnosis was the dependent variant instead of BMD at both femoral sites investigated [6]. Ozgocmen et al. determined that estimated cumulative steroid dose was negatively correlated with femoral trochanteric BMD, although there was no correlation with BMD at the femoral neck or lumbar region [27]. More recently, Tyblova et al. reported a negative effect of steroid and disability on BMD [28].

In addition to several studies that failed to show an association with reduced bone mass and intravenous pulse high-dose methylprednisolone therapy in patients with MS [9, 23–26], Schwid et al. reported a significant increase in lumbar BMD but no change in femoral BMD in patients with MS treated with high-dose methylprednisolone pulse (1 g/day for 3 days) [24].

We determined a significant association between disability levels and BMD. In order to better explore this hypothesis we evaluated the presence of osteopenia and osteoporosis in the two patient subgroups: EDSS scores not affected by ambulation (EDSS < 5) and EDSS scores based on limited ambulation (EDSS ≥ 5). We observed decreased BMD in fully ambulatory patients with MS. However, patients with impaired ambulation exhibited more prominent and a high proportion of decreased BMD.

Sioka et al. determined no association between axial BMD and neurological disability quantified by EDSS [6]. Similarly, Mojtahedi et al. determined such a correlation in a study of 29 female patients with EDSS less than 5.5 [29]. On the other hand, in parallel to our findings, the vast majority of previous studies have reported negative correlations between BMD and EDSS [7, 10, 24, 26, 27, 29]. The discrepancy between the results may be due to the different characteristics of the populations investigated.

A negative association was observed between duration of MS and BMD. Similar results have been reported in other studies [6, 9, 30].

There was a negative association between smoking and BMD, again in agreement with previous studies [31].

These effects may be mediated directly by toxic effects on osteoblasts in addition to the negative effects on bone vessels and nerves [31].

We determined no association between vitamin D and bone status in MS. However, one of the limitations of our study is that it was not designed to assess vitamin D and additional humoral or endocrine factors which may contribute to bone loss in patients with MS. Previous studies have reported an association between vitamin D status and low BMD [7, 19, 32, 33]. However, other investigators have reported no relationship between vitamin D levels and BMD in patients with MS [8, 10, 25, 27]. Another limitation of our study is that we included heterogeneous groups of different types of MS. Low frequency pulses and sporadic glucocorticoid pulses 1-2 times in a year may not have been a problem on bone metabolism in young patients. However, steroids can have adverse effects in patients with PPMS and SPMS in older MS patients.

## 5. Conclusion

Our findings reinforce the idea that motor disability and limited mobilization appear to be a major contributor to secondary osteoporosis in patients with MS. Pulse methylprednisolone treatment for relapse does not further exacerbate bone loss. We therefore suggest that it is not necessary to exclude relapse treatment with pulse dose methylprednisolone due to concerns over bone loss. The main clinical implication of our results suggests that physical rehabilitation and exercise programs for patients with MS are essential for better bone health.

## Figures and Tables

**Table 1:** Descriptive characteristics of MS patients and controls (mean ± SD or count).

	MS patients	Controls	*p*
Sex (female/male)	155 (104/51)	90 (58/32)	0.66
Age, all	35.6 ± 9.3	36.6 ± 8.5	0.36
Female	35.1 ± 9.1	37.7 ± 8.2	0.07
Male	36.7 ± 9.8	34.6 ± 8.7	0.32
Smoking years	10.7 ± 9.8	10.2 ± 9.4	0.44
Body mass index (kg/m^2^)	23.6 ± 3.2	24.1 ± 3.1	0.37
Mean age at onset	27.7 ± 8.1		
MS duration (years)	7.7 ± 5.8		
Relapse number	3.8 ± 2.9		
EDSS	2.6 ± 2.0		
Total steroid dose (gram)	13.9 ± 13.4		
25(OH)D (ng/mL)	12.4 ± 7.1		
RRMS/SPMS/PPMS%	71/26/7		

SD: standard deviation; RRMS: relapsing-remitting multiple sclerosis; SPMS: secondary progressive multiple sclerosis; PPMS: primary progressive multiple sclerosis.

**Table 2:** Comparisons of bone mineral density measurements (mean ± SD) at lumbar and femoral sites between MS patients and controls.

	Female			Male		
	MS patients *n* = 104	Controls *n* = 58	*p*	MS patients *n* = 51	Controls *n* = 32	*p*
Lumbar spine						
BMD	0.98 ± 0.15	1 ± 0.13	0.27	0.99 ± 0.17	1.00 ± 0.13	0.81
*T* score	−0.56 ± 1.37	−0.40 ± 1.11	0.47	−0.81 ± 1.51	−0.77 ± 0.87	0.89
*Z* score	−0.33 ± 1.37	0.06 ± 1.17	0.07	−0.68 ± 1.50	−0.57 ± 0.85	0.69
Femoral neck						
BMD	0.81 ± 0.17	0.87 ± 0.14	0.021	0.89 ± 0.23	0.92 ± 0.14	0.48
*T* score	−0.82 ± 1.59	−0.24 ± 1.35	<0.001^*∗*^	−0.67 ± 1.69	0.69 ± 1.53	<0.001^*∗*^
*Z* score	−0.48 ± 1.63	0.45 ± 1.43	<0.001^*∗*^	−0.05 ± 1.59	0.79 ± 1.46	0.018^*∗*^
Femoral trochanter						
BMD	0.64 ± 0.13	0.68 ± 0.10	<0.001^*∗*^	0.74 ± 0.16	0.71 ± 0.15	0.46
*T* score	−1.02 ± 1.20	−0.47 ± 1.15	0.005^*∗*^	−0.76 ± 1.29	0.32 ± 1.53	<0.001^*∗*^
*Z* score	−0.87 ± 1.19	0.15 ± 1.15	<0.001^*∗*^	−0.49 ± 1.23	0.50 ± 1.41	<0.001^*∗*^

SD: standard deviation. *p* values are from Student's *t*-test and ^*∗*^ *p* values <0.05 are statistically significant.

**Table 3:** Multiple regression analyses of BMD.

	Lumbar spine		Femoral neck		Femoral trochanter	
	Beta	*p*	Beta	*p*	Beta	*p*
Male/female	−0.019	0.840	0.239	0.080	0.247	0.077
Age	0.118	0.252	0.240	0.016	0.141	0.161
Disease duration	0.037	0.737	0.020	0.852	−0.087	0.418
Smoking years	−0.192	0.051	−0.223	0.019^*∗*^	−0.191	0.049^*∗*^
EDSS	−0.135	0.176	−0.270	0.005^*∗*^	−0.213	0.030^*∗*^
Cumulative steroid dose	−0.063	0.638	−0.127	0.327	−0.073	0.581
Relapse number	0.065	0.645	0.092	0.497	0.097	0.484

^*∗*^ *p* values <0.05 are statistically significant.
